# Réflexions Sur L’Intermittence Considérée Chez L’Homme Dans L’État De Santé Et Dans L’État De Maladie, &c.

**Published:** 1857-09

**Authors:** 


### NORTH AMERICAN MEDICO-CHIRURGICAL REVIEW.

SEPTEMBER, 1857.

### Analytical and Critical Reviews.

ART. I.—*Réflexions sur l'Intermittence considérée chez l'homme dans l'état de Santé et dans l'état de Maladie, &c.* Par E. PALLAS, M.D., &c. Paris. 8vo.

*De la Périodicité des Fièvres Intermittentes et des Causes qui la Produisent.* Par M. F. M. AUDOUARD, D.M.M. Paris. 8vo.

*Des Irritations Nerveuses sous le Rapport de la Thérapeutique.* Par GUERIN DE MAMERS, D.M.P. (Ann. de la Médecine Physiologique. vol. 8, p. 475, &c.)

*Des Fièvres Intermittentes sous le Rapport de l'Etiologie et de la Thérapeutique.* Par GUERIN DE MAMERS, D.M.P. (Journal des Progrès des Sc. Méd., N.S., vol. 2, p. 55, &c.)

*Traité Anatomico-Pathologique des Fièvres Intermittentes Simples et Pernicieuses.* Par E. M. BAILLY, D.M.P. Paris. 8vo.

THE theory of periodicity in its application to malarial fevers; in other words, the question of the circumstances by virtue of which the febrile paroxysms, constituting by their assemblage fever of the intermittent type, as well as the exacerbation of like excitement, forming, when separated from each other by intervals of marked though incomplete repose, remittent fever; and again, when those intervals, though returning at more or less regular periods of time, are slight or scarcely perceptible, and present themselves in the guise of pseudo-continued fever, is one of no small interest, and has attracted the attention of pathologists from an early period of our professional history.

By some writers a distinction has been drawn between periodicity and intermittence. The former is defined to be the recurrence of certain phenomena at either regular or irregular intervals, which are constantly reproduced by the renewed presence of the cause on which they originally depended; phenomena which would necessarily cease, were the same cause, or one capable of producing similar effects, never to return. By intermittence, on the other hand, is understood a succession of similar or nearly similar phenomena, regular or irregular in their periods of activity or repose, not resulting from the repetition of influences from without, but from some functional peculiarity inherent in the organs themselves.* Proper as the distinction may be in many respects in its application to physiological and pathological phenomena generally, it is doubtful whether it would serve a useful purpose in reference to the disease more particularly before us, inasmuch as though the latter does not require, for its continuance or repetition, the renewed presence of the cause on which it originally depended, yet it does arise exclusively from a functional peculiarity inherent in the organs themselves. For this and other reasons, the two terms have been very generally, and will, in the following pages be, used indiscriminately.

This phenomenon of periodicity, which occupies an important position among the distinguishing characteristics of autumnal or malarial fevers, and from the manifestation of which by these, to a greater or less extent, is founded the designation of periodical assigned to them everywhere, is certainly one of an extraordinary nature. It is one which many medical inquirers have regarded as not a little difficult of explanation, whatever be the view adopted respecting the pathology of the disease; whether we regard the latter as the effect of a malarial intoxication, or whether we admit it, as some have done, to be the result of a local derangement of some one or more particular organ or tissue, produced by the influential agency of ordinary morbific causes. For, say they, if periodic fevers depend on the alteration of the blood brought about by the absorption of a poisonous matter floating in the atmosphere, as believed by malarialists, we cannot comprehend how it comes to pass that the combination of symptoms characterizing the disease does not show itself uninterruptedly; in other words, why the fever fails to assume the continued type. Still more naturally should we anticipate this result, if the disease depended on the inflammation of some organic apparatus; inasmuch as intermittence and inflammation would seem to be antagonistic terms, the latter morbid condition being always continuous.

Without stopping to inquire how far the second of these propositions is

* Cowan. Trans. of Provincial Med. and Surg. Assoc., iv, 264, 5.

founded in fact, and without denying the difficulty of accounting for the phenomenon in accordance with either view of the pathology of the disease, it may be stated, that the occurrence of periodicity in the fever in question, is not incompatible with a belief in either the toxical or the inflammatory origin of the disease, since other complaints undoubtedly resulting from the absorption of poisonous matter, often assume the periodic type, and others in which the existence of topical inflammation, acute or chronic, is satisfactorily made out,—in which, indeed, that morbid state is evidently the starting-point in the chain of morbid actions,—exhibit the same phenomena of periodicity as fully and conspicuously as paludal fevers themselves. The question, then, is not whether the periodicity of those fevers is compatible with any particular view we may adopt in relation to the pathology of the disease, but,—admitting the extraordinary nature of the occurrence of temporary cessation or diminution, and return and aggravation, at regular periods of time, of the febrile symptoms,—endeavor to discover the influential agencies which give rise to those effects.

Be this as it may for the present, the periodicity of intermittent fever, and to a certain extent, if not as fully, of other complaints in which the phenomenon manifests itself, does not apply to the succession of the paroxysms alone, but is extended to the periods of repose intervening between these. It shows itself in the phenomena of relapse, of crisis, and of latency. Thus, relapses generally observe a regular order in regard to the period of their occurrence. The observation is of old date. Celsus observes, that when the fever ceases, we must long bear in mind the day of the paroxysm, and on that day avoid exciting causes. In our own time, Dr. Graves, of Dublin, ascertained that a quartan ague returned, after various lengthened periods, precisely on the day and hour on which the fit would have been due had the disorder progressed uninterruptedly. Kindred observations are recorded by Lucadou, Corbin, and many others. The disposition to a recurrence on that day, and indeed at the same hour, is retained for a long time. Van Swieten, in his Commentary on the 757th Aphorism of Boerhaave, tells us of a case in which it manifested itself five months after the cessation of the fever. Strack cites several remarkable cases of the same kind, and states that neither time nor change of climate, nor the distance from the infected locality, occasions any change in the order or day of the attack. Dr. Martin, who resided many years in India, and whose work on the diseases of tropical climates is now well known, says on this subject: "When the intermittent fever has been severe and long-continued, the disposition to recurrence of the same disease seems to last for life. After a residence of ten years in England, I happened to pass three nights at the best hotel in Strasburg, at a time when ague prevailed in the garrison amongst the French soldiers who had served in Algeria, and two days after quitting that town I was seized at the accustomed hour of 11 A.M., with ague, and I was the only person of the party who was so affected." Dr. Martin, after remarking that agues which have become habitual by repetition, impart a character of periodicity to other ailments, adds, that after his return to Europe, he became subject to facial neuralgia, which came on regularly at 11 A.M., the very hour at which ague used to commence with him in India more than twenty years previously. (P. 189.)

We know that in the form of fever, which, from its constant return at stated times, after apparent convalescence, has received the name of the relapsing fever, the symptoms, after the critical sweat of the fifth or seventh day, generally return on the fourteenth; and those relapses recur not only once, but several times. Other fevers, in like manner, show a disposition to recur at stated periods. In the government of Ufa (Russia), autumnal fever, which in that section of country is very common, attacks the patient every seventh day only, and is so severe that it generally proves fatal.

We cannot enlarge on the subject in this place, and must dismiss it with referring, besides the writings of the authors already cited, to those of Werlhoff, Grimaud, Nepple, R. Jackson, Balfour, Meade, Boyle, Johnson, Goodeve, Floyd, Murray, Coolidge, Dickson, Drake, for facts more or less apposite to the question under consideration.

In both his works, on Pneumonia and Malaria, and on the Yellow Fever, Dr. La Roche, of this city, has dwelt somewhat at length on the tendency to periodicity observed in the process of latency or incubation of malarial and other toxical complaints. The result of observations made during a period of a third of a century, have inclined him to the belief, which others had entertained long before, that the process in question in autumnal fevers generally, is ordinarily governed, as regards duration, by certain definite laws, analogous to those which preside over the progress, fluctuations, and return of the same and some other diseases. The late Dr. Robert Jackson was of opinion that the aptitude to receive the morbific impression of the cause of fever more at particular periods than others, that it manifests itself more frequently about the fourteenth day after communication with an infected source, and that it is observed chiefly at septenary periods, the seventh, fourteenth, twenty-first, &c., from the time of exposure. This opinion was based upon the results of his own observations "made upon numerous bodies of men, upon healthy men placed as attendants in infected hospitals, and upon healthy soldiers sent to concentrated sources of endemic fevers." Among such the fever scarcely ever appeared before the seventh day, commonly not before the fourteenth, and in numerous instances not till the expiration of six weeks, or even two months; though the cause of disease during the time was ordinarily in great activity. A distinguished writer of our own country, Dr. Dickson, of Charleston, has, in like manner, pointed out the fact. "The apparent influence upon it (fever) of the septenary revolution is familiarly noticed in our climate, where the opportunities for observation are unfortunately distinct and frequent. Our country fever is expected to invade on or about the fourteenth day, and if the twenty-first pass without an attack, most persons consider themselves entirely safe."*

Nor has it failed to be noticed that intermittent fevers exhibit during their course a tendency to a septenary revolution, and that at those periods, either after the seventh, fourteenth, or twenty-first paroxysm, they have a disposition to terminate spontaneously. This was frequently verified in Florida, under Dr. Forry's observations, and has been noticed sufficiently often elsewhere to justify its being received in the light of a well-established fact. In remittent fever, the same tendency is noticed, the disease having a particular disposition to a favorable critical change on the seventh, fourteenth, twenty-first, and twenty-eighth day.

In reference to the cause of the phenomenon of periodicity in malarial fevers and other diseases, medical writers have, from an early period, exercised their ingenuity. So far, it is true, the results obtained towards solving this intricate question have not been of a fully satisfactory kind. Nevertheless, sufficient has been obtained, and the inquiry has elicited enough ingenuity to render the subject one of no small interest to professional inquirers. Entertaining this belief, we cannot but think that a few pages devoted to the enumeration, examination, and analysis of the principal opinions that have, at times, been suggested on the topic in question, will prove acceptable to the reader.

By some of the older writers the phenomenon is attributed to the debility or the particular disposition of the affected parts, or to the qualities of such of the four humors as were supposed to be vitiated in the complaint. To the pituitous, as being the coldest and most viscous, the longest intervals were referred. The yellow bile gave rise to a paroxysm every third day; while the melancholic humor aroused the fever every fourth day. At a somewhat later period, Sylvius attributed the phenomenon to the introduction of the pancreatic juice into the blood. Borelli thinks it owes its origin to the successive irritation of the nervous extremities, of the nerves themselves, of the brain, and of the moving fibres of the heart; the whole resulting from an acidity or acrimony developed in the nervous fluid. Reil is of opinion that the intermittence of the vital actions is linked to that observed in the whole universe, and is the expression of a general law of our existence.

* Elements of Med. 2d ed. p. 179.

Ackerman, Ruediger, and others, refer the exacerbations in fevers and other diseases, and the phenomenon of periodicity generally, to certain accumulations of imponderable fluids (*aura oxygenea*) in the nervous ganglia, and to their discharge therefrom at given periods. Von Hoven finds the explanation in the natural activity of our organs when they are viciously affected by disease. Willis explains the phenomenon by the periodic development of a fermentable matter in the blood, and tells us that the fever intermits because all the morbific matter is expelled by a single paroxysm, and that until new matter is formed the state of apyrexia necessarily continues. Werlhoff, in turn, finds the explanation in the periodic movements of the globe. Meade, Balfour, Jackson, Gillespie, and several other more modern writers, following in the footsteps of Galen, refer the phenomenon to lunar influence; Meade adding the alternate action of night and day, the direction of the winds, as also the agency of the equinoxes and solstices. Stalh and several more have recourse to the power of habit,—not without admitting, however, that in many instances the cause is inscrutable. Gemma discovered the cause in the inferior position of such of the organs as he supposed to be affected in periodic diseases.

"In every fever," says Cullen—whose views on the subject are not very different from those of Bryan Robinson—"in every fever in which we can distinctly observe any number of separate paroxysms, we constantly find that each paroxysm is finished in less than twenty-four hours; but as I cannot perceive anything in the cause of fevers determining this, I must presume it to depend on some general law of the animal economy. Such a law seems to be that which subjects the economy in many respects to a diurnal revolution. Whether this depends upon the original conformation of the body, or upon certain powers constantly applied to it, and inducing a habit, I cannot positively determine; but the returns of sleep and watching, of appetite and excretions, and the changes which regularly occur in the state of the pulse, show sufficiently that in the human body a diurnal revolution takes place. It is this diurnal revolution which, I suppose, determines the duration of the paroxysms of fevers; and the constant and universal limitation of these paroxysms, while no other cause of it can be assigned, renders it sufficiently probable that their duration depends upon and is determined by the revolution mentioned. And that these paroxysms are connected with that diurnal revolution appears further from this, that though the intervals of paroxysms are different in different cases, yet the times of the recession of paroxysms are generally fixed to one time of the day; so that quotidians come on in the morning, tertians at noon, and quartans in the afternoon."

On a subject of the kind, it was not to be supposed that Darwin would fail to offer an explanation or what he considered such. When—we use in part Dr. Craigie's summary—from any cause, the sensorial or nervous influence over the system is diminished, the motions of the vascular and other dependent systems are also impaired and enfeebled. During the state of quiescence or inactivity thus induced, however, sensorial power is accumulated, and as it was saved before, it now becomes excessive; and this takes place, he argues, soonest in the capillaries of the skin, which are most remote from the influence of the sensorial or nervous energy. The excessive development of sensorial power then takes place in the increasing action of the hot fit. "Now, this increased action of the system during the hot fit, by exhausting the sensorial powers of irritation and association, contributes to induce a renewal of the cold paroxysm, as the accumulation of those sensorial powers in the cold fit produces the increased action of the hot fit, which two states of the system reciprocally induce each other by a kind of libration (oscillation ?), or a *plus* and *minus* of the sensorial powers of irritation and association." Darwin further imagines that the cold fit produces a congestion of vessels in some of the organs, from which their vessels do not entirely recover in the hot fit, and hence, that every preceding cold fit must lay the foundation for a subsequent one.

Hildenbrand next attempted the explanation of this subject. He had recourse, with this view, to the periodical revolutions exhibited by cosmic phenomena, or the revolutions of the earth and moon, in other words, to Sol lunar influence. His theory, therefore, is little more than a modification of that of Meade, Balfour, and other writers, already referred to. In connection with this subject, he remarks, says Dr. Craigie, from whom we derive the reference, the periodical regularity observed by many of the phenomena of organized bodies, for instance, the alternate succession of sleep and watching, the periodical recurrence of the menstrual discharges, &c. Quotidian paroxysms, whether of intermittents or remittents, he represents to be under the influence of the daily rotation of the earth on her axis; those of the tertian, observing the first, third, fifth, and seventh days, and the quartan observing the first, fourth, and seventh, appear to correspond to lunar periods; while the duplicated and tripled tertians depend on the combination of lunar and telluric influences.

Bordeu suggested that the regularity with which the paroxysms return—often at fixed hours—might be due to the succession of organic movements, and to the greater susceptibility of action of certain essential viscera, the liver, spleen, &c, an action which may have regular periods, analogous to those of the uterus, of the salivary glands, of the generative organs in lower animals, and of other parts, with the periods of activity and repose of which we are but imperfectly acquainted.

According to Giannini, the phenomenon of intermittence is established because sensibility is considerably diminished during the sweating stage. On the increasing debility of the arterial system,—a debility capable of diminishing the circulatory movement of the blood, and of preventing the development of caloric,—depends the accession of the paroxysm, and "to the greater or less length of time which living systems require to reach the degree of debility and of depression in the calorific faculty necessary to the development of the paroxysms of intermittent fevers, must be referred the variety of their types. When this depression operates in one day, quotidian fever is the result. When two days are required, we have tertian fever; when three days are required, the disease assumes the quartan type. From this we may conclude that intermittent fevers, the type of which, in order to be developed, requires a long space of time, must be those which will retain with most force the morbid modification occasioned in the system by the course of the disease." *

Several other theories, or, perhaps more properly, hypotheses, have been offered, and deserve a passing notice. Virey, modifying the views of Stalh, attributes the effect to the influence which the circulation or rotatory movement, habitual in the human system and in that of most animals, exercises on the return of the functions. Agreeably to him, parts acquire, in a progressive manner, by the gradual flow of blood, and during a period of time regulated by habit, a certain amount of plethora and vital force. When this has reached its ultimate point, the parts are disgorged of the excess of blood, either in its pure state, or in the form of secretions, or some other such means, or else the nervous excitant is consumed or exhausted after manifesting itself in the form of pain or febrile irritation. Hence, the occurrence of periodic paroxysms of fever constituting periodic fever. Periodicity, therefore, according to this theory, "consists solely in the effect of the accumulation of certain morbific causes or of secreted fluids, which determine a relaxation, a discharge, a paroxysm, at certain periods more or less distant from each other, and more or less regular. In the same way the rhythm and the return of sounds relieves and sustains the strength in all operations of some duration, because it establishes intermissions or periodic reposes, which enable our faculties to rest and to recuperate their wasted strength. It is thus, also, that the quotidian returns of day and night, and of the waking state and sleep, preserve our existence, and renew continuously the play of our functions."†

Bailly, in his important and truly valuable work on Intermittent Fevers, calling attention to what he represents as well-established facts, that the lower animals are not subject to intermittent fever, and that, while in ma-

* Giannini, Della Natura della Febbri, i, 116, 117.

† Dict. des Sc. Med. lx. 428–9.

larious regions and during epidemics of periodic fevers, the human species suffers from the disease, the other experiences the effect in a different way, endeavors to explain the circumstance, and to account for the periodical character of the morbid phenomena in the former species, and its absence in the latter, by a reference to the nature of their respective positions. The constantly horizontal position of the lower animals, compared to that of human beings, which varies and alternates regularly night and morning from the vertical to the horizontal, must, as he confidently infers, exercise a marked influence in the development of the phenomenon in question. Thus, in reference to man, every morning, on his assuming the erect position, the circulation undergoes a change; the nervous system of the abdominal organs is excited into activity; the gastric organs are more highly stimulated, their functions are performed more energetically; in a word, they become the centre, starting-point, or focus of vital movements. Hence, all diseases which have their seat in these organs exhibit a morning exacerbation. In the evening, on the contrary, when man assumes the horizontal position, his brain becomes the centre of the vital movements, blood flows into it in greater abundance, and circulates through it with more activity. Hence, the paroxysms of diseases located in that organ occur at this time.

The morning excitation of the abdominal nervous system, and the evening excitation of that of the brain, constitute, in this view, two natural phases of a physiological function which is scarcely perceived in health, simply because it is habitual. When, however, from the operation of special causes, such, for example, as paludal exhalations, a greater degree of activity is imparted to this function, the result will be, that the morbid excitation produced in the abdominal organs will assume an intermittent character. "An intermittent fever is, therefore, the exaggeration of the combination of organic acts constituting a nycthemeron, and which take place in the following ways: 1st. Morning congestion of the stomach and intestines; 2d. Augmentation of the different nervous influences which are exercised on the whole economy, and which, according to the particular disposition of the individual, and to the causes above specified, give rise to a particular nervous symptom rather than to another; 3d. Cessation of the congestion by the horizontal position."*

M. Roche, a distinguished pupil of the School of Broussais, has set forth an hypothesis of a different kind. He thinks, 1st, that irritations which exhibit the intermittent character, are always prepared by causes the action of which is also intermittent; 2d, that the development of these irritations is favored in some organs by the intermittence of their functions; 3d, that they are almost always produced or excited into being by intermittent causes; 4th, that sometimes the persistence of these causes,

* Traité Anatomico-Pathologique des Fièvres Intermittentes, &c. p. 5–32.

sometimes the influence of habit, and often both of these agencies combined, tend to keep up those irritations; 5th, that intermittent irritations which are not due to the preceding causes, derive their periodical character from some accompanying circumstances. According to this writer, the predisposing causes of intermittent irritations are always themselves of an intermittent character. "Thus, spring and autumn are the periods of the year during which those affections are most frequently developed, and the character common to those two seasons, is to present a considerable difference between the temperature of day and that of night. These alternations of cold and heat keep up in the economy a continual alternation of action and reaction; hence the establishment of intermittence. Now, if in an individual thus modified, a stimulus happens to act on one or other of the organs, especially if the function of the organ is subjected, in the state of health, to the law of periodicity, the lesion will become intermittent. If to these periodic predisposing causes, is added the intermittence of the stimulus itself, there will necessarily be intermittence of the disease."*

Such is the theory originally suggested by M. Roche. According to it, the nature of the stimulus producing the disease is a matter of no consequence. Provided the system is predisposed by the action of intermittent causes, the effect will be an intermittent irritation, and under particular circumstances, an intermittent fever properly so called. The effect will be more certain to result, if the stimulus which excites the disease into being is itself intermittent. Since the publication of his essay, the views of M. Roche have experienced important modification. He now regards intermittent fever as the result of the intoxication of the blood by miasms. Each paroxysm, according to him, represents the four phases of the phenomenon of intoxication: 1st, malaise, indicating the near approach of the morbid state; 2d, contact of the poison with the principal organs; 3d, reaction of the economy against that agent; 4th, efforts at elimination. But each paroxysm does not imply a renewal of intoxication, for we find the fever recurring again even when the patient has left the infected spot. Nevertheless, it is probable that the elimination of the miasm is not completed before a certain number of paroxysms, proportioned perhaps to the degree of saturation which the patient has undergone. It is perhaps in this series of incubations, corresponding to a series of efforts at elimination which are necessarily repeated so long as the morbid agent has not been effectually expulsed from the system, that we are to seek for the explanation of the phenomenon of intermittence. While admitting this, however, M. Roche continues to view the intermittent action of the causes, as also the power of

* Ann. de la Med. Physiol., i, 116, &c. 1822.

habit as exercising the principal influence in the production of the phenomenon in question.

M. Guérin de Mamers, another disciple of the School of Broussais, but who, unlike many of the admirers of the great pathologist, presumed, on several occasions, to think for himself, is of opinion that the paroxysms and intermissions of periodic, as well, indeed, as the exacerbations of continued diseases, must be attributed to an extraordinary development of the nervous influence, to its concentration on a given point, and, subsequently, to its reproduction, as well as to a recurrence of transient congestions. For, according to him, the only difference which exists between complaints accompanied with simple exacerbations and those presenting complete paroxysms, consists in the circumstance, that in the former the nervous irritation is complicated with permanent vascular irritation; while in the latter it is accompanied with a vascular congestion as transitory as itself. Refusing to admit the possibility of a permanent inflammatory congestion giving rise to fever of a periodic character, he affirms, that the intermittent type is the exclusive attribute of affections of the nervous system, and that it appertains to the functions of that system. Intermittence has its real cause in the economy itself, and in a more especial manner in the nervous system. Hence it is a phenomenon of a perfectly organic, and, as a consequence, of a physiological character.

In reference more particularly to the periodicity of intermittent fever, Dr. Audouard, who claims to be the originator of the splenic doctrine of the pathology of the disease, suggests the following views. Intermittent fever, according to him, is caused by a peculiar agent, which being introduced into the body, modifies the circulation of the blood by altering the nature of that fluid. This agent is a poison endowed with more or less activity, and originating from the marshy exhalations; in other words, from the putrid decomposition in great part of vegetables, and of insects that perish in the mud. This modification of the circulation of the blood possesses a somewhat thermometric character. It is subordinate to the heat of the climate, and to the diurnal solar influence. The diurnal and nightly intermittences, accelerate or retard the action of the vascular system, and the congestions of blood are proportioned to the solar influence, modified by the idosyncrasy of the individual. The spleen, which acts as a reservoir of the blood, is the seat of this congestion, which would rise to the state of inflammation were this organ susceptible of taking on this morbid process. But from this alternate action of the heat of day and coolness of night, there arises a derangement which is in like manner alternating in regard to its phenomena; in other words, an intermittent fever. The diurnal solar influence, he continues, exercises a positive action on the economy. This action might be mathematically demonstrated, if all human conditions and constitutions were alike. In the absence of this uniformity, we may say that it is relative. If, on a given day, the malarial action has not been sufficiently active, or has not encountered an individual sufficiently excitable, to produce on him a splenic congestion of a character capable of determining a paroxysm, the latter does not take place; and during the following night, the circulation of the blood, to which the spleen contributes, being no longer under the positive influence of the sun, diminishes the congestion which had been formed in that viscus, without, however, removing it entirely. But the solar influence of the next day, adding to what remains of the congestion of the day before, carries it to the degree it must attain, in order that it may occasion the general derangement or paroxysm. From this arises a tertian fever. A similar reasoning is applicable to the other types. Thus, during the warmest and longest days, quotidians are due to a solar action capable of producing a splenic congestion of sufficient intensity to give rise to a daily fever. On the contrary, in the commencement of winter, the days being shorter and the solar heat less considerable, its action is less intense, and occasions a general derangement or paroxysm every third day.

As may be already known to most of our readers, several writers have, in imitation of Audouard, placed the seat of periodic fever in the spleen, and attributed to that organ no inconsiderable share in the production of the phenomenon of periodicity,—Piorry; F. A. Durand, of Lunel; S. Durand, of St. Aubin, &c. According to the first, who alone claims our attention on this occasion, a paroxysm of periodic fever consists in an attack of progressive neuropathy, which starts from the abdominal or thoracic plexuses,—from those of the spleen, kidneys, and genital organs particularly. This neurosis spreads successively to various points of the cerebro-spinal apparatus. Starting from the nervous plexuses, it ascends towards the centres, and finally extends to the circumference. It is reproduced towards the skin, and causes chills, in the same way that we see various neuralgias, uterine and others, repeating themselves towards various points of the nervous system. 2d. Paroxysms of fever which are renewed a limited number of times, and in a periodic manner, may originate in the renal, spermatic, ovarian plexuses, &c. In such cases, the neuropathia extends from those plexuses to the splenic nerve. 3d. Periodic paroxysms,—quotidians, tertians, quartans, &c., have their true starting-points in the ramifications of the splenic plexus. 4th. Various lesions of the spleen may give rise to that neuropathia. 5th. Legitimate cases of intermittent fever most generally arise from splenemia, combined with increased volume of that organ. 6th. This lesion is most generally produced by the action of paludal miasmata. 7th. The first effect of these miasmata is a direct action on the blood, from which there results a true toxemia. 8th.

The effect of this alteration of the blood is to act on the spleen, and to cause the engorgement or hypertrophy of that organ, and as a result of this, the periodic neuropathia which characterizes an intermittent fever. 9th. In its turn this splenopathia produces a particular action on the blood, manifested by the special complexion, the general debility, and other changes noticed in individuals long affected with periodic fevers.

It must follow from this, if correct, that periodic fevers have their origin, or their cause, in the engorgement of the spleen, and that, hence, to say splenic engorgement is equivalent to saying periodic fever; and, reciprocally, to say periodic fever is equivalent to saying splenic engorgement. So intimately are the two,—engorgement and fever,—linked together, in the estimation of Piorry, that we may suppose that if man had been constructed without a spleen he would be spared all the inconveniences resulting from periodic fevers.

A writer of our own country, Dr. Clay Wallace, is of opinion that until more satisfactory data are furnished, he will assume that intermittent fever is caused by inhaling the spores of a fungus growing on decomposing organic matter; that these spores are received into the circulation and deposited on branches of the sympathetic; that the periodical development of these deposits occasions the chills and vascular reaction; that the symptoms cease when the nerves are accustomed to the foreign body, and are again renewed by another development, which, in tertians, occurs every third day, just as spores have been observed to form always on the fourth day in the fungus of muscardine.* Another writer, Dr. Wood, of this city, takes a very different view of the subject. After remarking that it is a law of our physical as well as moral nature, that excitement, even under the constant influence of stimulating agencies, cannot be long sustained at a particular point of elevation, and that the change of remission and exacerbation usually takes place once in twenty-four hours, Dr. W. states that the cause of this is probably to be found in that habit of the system acquired by the daily alternation of waking and sleeping. The excitability of the former condition being regularly diminished in the latter, a flux and reflux is established, which, though in cases of disease it may occasionally vary in the precise point of accession or decline, is still, as a general rule, experienced within the habitual limits. Upon the same principle, to a certain extent, he continues, may be explained the disposition observed in intermittent diseases to a daily return of the paroxysm. The cause of the disorder gives rise to the first paroxysm, at that period of the twenty-four hours at which, in the advance or the recession of the excitability, the system is in the most favorable condition for its action. The same condition is experienced about the same time on the following day, and the same

* Boston Med. and Surg. J., Oct. 17th, 1847. Charleston J., x. 778.

result necessarily takes place, if the cause continue to operate. That the paroxysm should often occur every other day, and sometimes every third day, as in the tertian and quartan agues, must be referred to some unknown modification in the character of the cause, or in the properties of the system, which prevents the equilibrium of health from being disturbed on the first favorable occasion, and enables the vital resistance to triumph over the morbid influence, till, weakened by successive attacks, it is compelled at last to give way.*

From what precedes, the reader can scarcely have failed to perceive that many of the hypotheses heretofore presented on the subject before us, are little more than modifications of each other. In many cases, the authors, while aiming at originality of views, and making a display of learning and ingenuity, have rested their conclusions on bare assumptions, and appealed to facts and statements of more than doubtful authenticity. They have exhibited, besides, a great deficiency of correct physiological knowledge. That some of these theories are plausible enough, and embody, or are to a certain extent founded on, facts of undoubted truth, and of an apposite character, may be admitted; but, whether plausible or otherwise, there can be no doubt that none are calculated to be viewed as satisfactory by the well-informed and careful medical inquirer. Some, indeed, are visionary in the extreme; others may even be said to be absurd, while others again can hardly serve a greater purpose than to assist in the explanation of the phenomenon in question; while from not a few we reach the sage conclusion, that the thing is so because the Maker of our being has willed it, and it cannot be otherwise; or the phenomenon is explained by the statement of certain circumstances, which themselves are left unaccounted for. In a word, the majority of the explanations suggested,—those of Sylvius, Borelli, Reil, Ackerman, Ruediger, Willis, Stahl, Werlhoff, Darwin, Meade, Balfour, Hildenbrand, Giannini, Virey, Guérin de Mamers, Bordeu,—must be rejected completely, or noticed only as belonging to the domain of medical history, or as constituting objects of mere curiosity. They teach nothing useful. They are either based on scientific principles long since exploded, or fanciful notions which cannot stand the test of examination, or on assumptions equally difficult to make good, or again on false interpretations of well-known facts. In some instances they do little more than state the occurrence of long-observed circumstances in different terms from those in which they are usually described; or, finally, while attributing the results exclusively to the influence of certain agencies, they take no notice of other agencies of as potential a character as the former.

Nor are the views of Cullen, Bailly, Roche,—the most talked about in

* A Treatise on the Practice of Medicine, i, 184. 1st Ed.

modern times,—free from serious objections. Cullen may have discovered a part of the truth, when he ascribed the periodicity of fever to some law of the animal economy, whereby it is subjected, in many respects, to a diurnal revolution. But he certainly did not discover the whole truth, and, after all, only stated the fact without explaining it. In a well-ordained and complete theory, it would be necessary to explain the cause of this diurnal revolution, which Cullen refuses to do, and to show how far the phenomenon in question depends upon it, and whether they are not both the effect of some other and antecedent cause. Besides, Cullen, as Dr. Watson well remarks, is much perplexed with the differences of type, and all that he can say on that point amounts to this, that as the three principal types observe, severally, a particular time of day for their accession, and as quartans and tertians are apt to become quotidians, these to pass into the state of remittents, and these last to become continued; and that as, even in the continued form, daily exacerbations and remissions are generally to be observed,—all this marks the power of diurnal revolution.

M. Bailly's theory is based, in great measure, on erroneous statements, and false physiological and pathological premises. It is not true that the constant horizontal position is a safeguard against periodical fevers, and that the latter are the attributes of human beings in consequence of their daily and frequent change of posture,—from the horizontal to the vertical. In stating, in support of his views that the lower animals are exempt from periodic fevers, M. Bailly has advanced an opinion too easily disproved to require a detailed notice in this place. Facts of an opposite kind are mentioned by Dupuy, Clichy de Janville, Leblanc, Schnurrer, Boudin, as occurring in various domestic animals,—dogs, horses, sheep, &c. The cause of the disease affects individuals who are lying down, and from sickness or other causes do not assume the erect position. The diurnal revolution marking the periodical excitement of important gastric and other organs, takes place whatever be the position in which the body is placed. It may be more marked when the body is erect, and especially when the latter is in motion; but it invariably comes on. In fact, the change of position cannot act on the nervous and circulatory functions otherwise than in a physical manner, and consequently too feebly and limitedly to change the order of the vital movements; and it is evident that the alternate excitation of the functions of the two systems of nerves is an attribute of life itself, and is linked to the primitive movements of ensemble or harmony, which regulate the action of each organ and apparatus. These movements are of too important a character to depend on the position which the individual may choose to assume.

Nor is it true, as asserted by M. Bailly, that febrile paroxysms invariably occur in the morning. It is a fact sufficiently well ascertained to require no illustration in this place, that such is not the case. Intermittents,—quotidians or tertians,—especially when complicated with gastric or gastro-hepatic irritation, almost always commence, in summer, from eight to ten in the morning. In remittents, the exacerbations occur after the hour of noon. The longer the fever continues and the less violence it presents, the more protracted the moment of attack becomes. The accession of fevers of the quartan type takes place from twelve to four o'clock; the attack, in some cases, being retarded, in others anticipated without the occurrence of any difference in the symptoms, capable of enabling us to explain or foresee the anomaly. Let it be remarked, that the abdominal nervous function spoken of by Dr. Bailly is, as has been well remarked by Dr. Nepple (288), neither tertian, double tertian, nor quartan. It is exclusively and constantly quotidian. Such being the case, it is difficult, admitting the views laid down by the former writer to be correct, to explain the diversity of types assumed by the disease. To this again it may be added, that Dr. Bailly has not attempted to apply his theory to several varieties of periodic fevers. If the human species acquires a predisposition to those ailments in consequence of gastric excitation and congestion resulting from the erect position, and while acquiring a tendency to cerebral disease, loses the other by assuming the horizontal position, it ought to follow that the lower animals, that are supposed to owe their exemption from the fevers to the latter position, which they constantly preserve, ought to be very liable to cerebral congestion,—to a constant semi-apoplectic existence. And yet we do not observe this to be the case. Finally, while malaria is supposed to act by merely exaggerating, in a pathological way, a condition of things which, in the state of health, would escape observation, and though it is only thought to be a stimulus, and to produce an intermittent fever in consequence of the gastric organs being predisposed to its morbid action, owing to the excitation produced in them by the vertical position of the body, we find that ardent spirits, ether, musc, and a variety of other stimuli, and even the paludal poison when in a state of concentration, produce disorders of a continued or pseudo-continued type.

In reference to the theory suggested by M. Roche, it may be remarked, that the causes which he regards as predisposing the organs to an intermittent action, are not more apt to produce that effect than many others. Atmospheric changes, it is true, are more frequent in the spring and latter part of winter than at any other time; but they are not more regular or periodic. Nor can we say that a great irregularity in the intermittence of the causes is capable of occasioning a regular intermittent inflammatory action. Indeed, we do not find intermittent fevers abounding at that season of the year. In winter, we are subjected to frequent and opposite changes of temperature. Most of the influential agencies to which we are exposed act in a periodic manner, as aliments, exercise, sleep, cold, heat; while the influence of malaria, though more active during the night, is, perhaps, less periodic in its action on the economy than the preceding. Besides, at that time a majority of individuals are sheltered from the influence of the poison, and if attacked, are so but seldom during the night. The temperature of autumn—August, September, and October—when fever prevails most abundantly, is less variable than stated by M. Roche. Evening may be damp, but is not everywhere cold; and, as remarked by M. Nepple, there is every reason to believe that this humidity plays a more important part in the causation of fever than the intermittence of action of the causes of the disease. Cold succeeding to heat much more frequently gives rise to local inflammation than to intermittent fever, which is much more frequently preceded by the stimulation of heat and excessive fatigue. If the intermittence of action of a morbific cause could communicate to the organic acts an analogous type, all diseases would assume that type, for there are few causes to which the speculations in which M. Roche indulges may not with equal propriety be applied.

On the other hand, M. Roche is not justified in attributing much influence to the intermittent functions of the organ upon which the stimulus must operate to produce periodic diseases. Few organs manifest a greater intermittence of function than the brain; and yet, no phenomena are less intermittent than those produced by alcohol, even when taken periodically. As to what M. Roche says in reference to the fact, that the paroxysms are repeated sometimes through the influence of habit, sometimes in consequence of the repetition of the cause, and often from the combination of both agencies, we need say little. According to him, habit consists in the tendency which all our organs manifest to repeat certain acts, though the causes from which they originally arose have ceased to operate. But habit thus defined does not explain the phenomenon. It merely expresses a fact, but it is not certain that that fact can occur in connection with febrile intermittence. In conclusion, it may be remarked, that it seems to be a very serious objection to M. Roche's theory, that the disease does not show itself, sometimes for weeks or months after the patient has been exposed to the malaria, that it occurs occasionally at a considerable distance from the infected locality, and that it is brought on by exciting causes of a diversified, though not necessarily of an intermittent kind. The theory fails also to account for the difference existing in the types of periodic fever.

But it would be vain to expect success in the investigation of the cause of the phenomenon in question. Periodicity may be regarded in the light of a primordial fact, of which we should study all the conditions without troubling ourselves with the proximate cause any more than we do with that of other modes of existence. Assuredly there is no propriety in asserting that intermittence is more inexplicable than continuity; for so long as existence itself shall continue to be an insoluble problem, no one mode of existence can be held as more inexplicable than the other. "In good philosophy we must study, collect, group together and combine facts. The imagination alone fills up the gaps; and it is that which is called to explain." Periodicity, then, is the result of the exercise of the same great law which regulates other phenomena manifested in health and disease—relapses, incubation, critical efforts, &c. The intermittent is as much a natural type as the continued. Need it be said that it characterizes many of the phenomena of health, and exhibits itself in the physiological play—both as regards progress and intensity—of many of the functions; in the processes of secretion, elimination, and calorification; in the operations of the nervous system; in muscular contraction; in the action of the heart, &c.? Intermittence, indeed, may well be viewed as an element essential to the existence of the normal actions of the economy. It does more; it adheres to these actions in their passage from the state of health to that of disease, and may, therefore, be recognized as an element of this state also. It stands, as has been said elsewhere, as an illustration of the great law of periodicity which regulates all the vital movements. Can we wonder that this law should have elicited the attention of medical and physiological observers from an early period of our science, and should not have been neglected in modern? An American writer, the late Dr. Carpenter, of New Orleans, has, among others, pointed out this manifestation of intermittence in the functions, and after showing, as Dr. Laycock had done already, that the periodicity of these is governed by a power or agency inherent in the system, and only in a secondary manner dependent upon the physical influences which surround us, properly remarks: "This principle once established, it becomes an easy matter to account for the intermittence of disease, by referring it to the persistent and controlling influence of those physiological oscillations, whose periods and intervals continue to mark and measure its stages and paroxysms. The matter of surprise and inquiries will then become, not why some diseases are intermittent in their course, but rather, why it is that all of them are not so?"

In his treatise on General Pathology, Henle enters into an examination of the subject with his accustomed ability, and adopts views, both in reference to the periodicity of health and that of disease, not very different from those to which attention has been called. All pathological symptoms, he remarks, are only the phenomena of the physiological vital process under changed conditions. So, also, rhythm in disease is nothing more than the rhythm of the normal vital process itself. This in disease may become more distinct; it may become changed, obliterated, and even disappear entirely, as, for instance, in mania and other nervous diseases, every trace of the periodic exacerbation and remission of the nervous activity is often lost between the time of sleep and waking. But in every case, there are only two causes to which the periodicity of disease is to be referred: either the noxious potency operates rhythmically, or the morbid symptom depends upon the changed function of an organ whose healthy life is periodical.

In disease, as in health, he further remarks, the accidental rhythm is, in the first place, to be distinguished from the natural. Pains and spasms excited by permanent causes, of themselves abate from time to time, because the vital power of the nerves becomes exhausted. Even in pure inflammation, and still more in the complicated, the pains appear in shocks, or increase in violence by paroxysms. Upon the same principle, tetanic and hydrophobic spasms are intermittent. Convulsions, from pathological formations which irritate the brain and spinal marrow, and from inflammation of these organs, cease when, as it is said, the irritability is consumed. The intermittent character of reflected spasms is most striking. In the common catarrh, in croup, and hooping-cough, the inflammation is indeed continual, just as when a foreign body is lodged in the air-passages, but the reflex motions, namely, the cough, appear only after longer or shorter pauses, and in paroxysms; so, also, sneezing from taking snuff, vomiting from irritation of the mucous membrane of the stomach, and colic from inflammation of the bowels. So, also, even in the healthy body, labor pains, and the contraction of the bowel and bladder, for the purpose of expelling their contents, appear in paroxysms. But even here it is very difficult exactly to distinguish the accidental from the natural rhythm; because, if we assume that a short intermission of the urgency to stool or urinate is the result of a momentary exhaustion, it will still very often happen, that this unsatisfied necessity is entirely suppressed until the time of the next normal paroxysm.

Henle, in continuation, remarks, that it has been a question much agitated, whether there are any true continued diseases. This expression, he says, is erroneous, inasmuch as even the so-called rhythmical diseases are continued, and only the symptoms are rhythmical. The expression should much rather be, Are there morbid symptoms with true *typhus continens?* They are generally allowed where there are disorganizations. But even with these, it often happens as if the formation of the morbid product ensued periodically, more rapid and more slow, or even experienced a cessation of growth, although that which is once formed never disappears again. Only the residua of disease, as scars and others, are completely stationary. On the other hand, it is denied that the continued type can be applied to any of the so-called pure functional disturbances. He says so-called, because disturbances of function without changes of matter are not supposable. This also is not entirely correct. It is true that all symptoms dependent upon the nervous system, do not continue uninterruptedly in an equal degree of violence or strength; but this kind of periodicity is accidental, an alteration of excitement and exhaustion; on this account, pains and spasms, and even febrile symptoms, always appear in remissions and excerbations, although not always with true natural periodicity. There is, on the other hand, scarcely any one symptom that has not sometimes been observed with a regular rhythm, remittent or intermittent: congestions, inflammations, hemorrhages, fluxes, and retentions, eruptions, mental diseases, and even paralysis, appear at one time more or less continuous, at another in shorter or longer pauses.

Henle remarks further, that it is a question very properly suggested, whether the exacerbations and remissions of rhythmical diseases coincide with the natural exacerbations and remissions of healthy life, and are merely the consequences of the latter; or whether the periods of arsis and thesis, in diseases, are dependent on the normal one, and are perhaps conditioned by the time when the morbific potency first exerted its influence. If the first supposition is correct, the paroxysms of the same disease must appear in all patients at the same hour of the day or night; but, if the first paroxysm is occasioned by the influence of the morbific potency, and the succeeding paroxysms, repeated in definite intervals, are repetitions of the first, then, as the period of the external influence is accidental, the time of exacerbation of the same disease is also different in different patients. In relation to this subject, he has no doubt it may be asserted that all rhythmical diseases make their exacerbation regularly at definite periods of the day; while in other diseases, on the contrary, the periods of exacerbation or of paroxysm, even though they are repeated in each individual case exactly rhythmically, are still less fixed in reference to the time of the day.

Justified, however, as we doubtless are, in referring the phenomenon of periodicity, as exhibited by the fevers under consideration, to the operation of the general law above alluded to, and willing as we must be to content ourselves with marking the effects of that law without attempting to clear the mystery by which its proximate cause is shrouded, it might still be a question worthy of examination, why some external agencies in their operation on the living system impart to the diseases they occasion this character of periodicity more than other morbid influences as potent in their action; why the same agencies, under other, or even analogous circumstances, produce effects of a different kind; why causes which ordinarily occasion complaints unmarked by the phenomenon mentioned, at times give rise to symptoms subject to periodic returns; and why the influences to which the result has been ascribed,—diurnal revolutions, the flux and reflux, increase and decrease of excitation, alternations of temperature, alternation of waking and sleeping, the power of habit, &c.,—should not more generally produce it, or why they succeed in doing so only in certain localities, and in individuals particularly predisposed to the effect from exposure to specific toxical agencies. Intermittent diseases are sometimes excited into being by causes which ordinarily give rise to complaints of a different character. While such is the case on special occasions, periodic fevers are the usual products of agents of a special kind, which, though sometimes failing to produce the effect in question, are almost certain to exhibit their power to that effect on individuals exposed to their action. Persons unexposed to those agencies experience with perfect impunity all the modifying influences mentioned, or if they sicken, suffer from diseases of a different character. In view of all these circumstances, we must infer that there is something in the true or efficient cause of periodic fevers which arouses more effectually the tendency existing in the vital movement of certain organs or tissues to assume, when morbidly impressed, the periodic character. But what that something is, how it acts to produce the effect, and why it should do so more than other agencies, are questions to which, in the present state of our knowledge, it would be impossible to give a satisfactory answer.

But, while expressing this opinion,—while maintaining that all the diurnal revolutions, all the fluxes and refluxes, increase and decrease of excitation, all the alternations of temperature, or of the sleeping and waking states, &c., cannot alone account for the phenomenon in question, and that something more is required to produce the effect, we are far from denying to these influences some co-operating agency in the matter. There is no reason to doubt that morbid organic periodicity is a nervous phenomenon. It is an attribute of the nervous system, and its manifestation in other parts of the economy is the effect of a complication or the result of a condition of those parts, imparted to them by the nervous matter entering into their composition. Whenever it is present as a pathological condition, it may be regarded as indicative of a lesion, primitive or secondary, of a portion of that system; for it is only here, in the absence of disease elsewhere, that any traces of its existence can be detected. Every one knows the close analogy, if not the identity, of character existing between neuralgic complaints, properly so called, and intermittent or malarial fevers. We are aware that all local affections of nerves present periods of quiescence and activity; that the former of those sets of complaints, scarcely less than the other, are accompanied, often at least, by shivering and a sense of cold, followed by heat and perspiration; that where intermittent fever prevails these neuralgic affections are frequent also; that individuals of a nervous temperament are most liable to that species of fevers, and that both diseases are benefited by, indeed are under the control of, the same remedial means.

To which division of the nervous system the phenomenon in question should be referred, is a question that may admit of some doubt. That periodicity is an attribute of, or is located in, the ganglionic system alone, or that it may be traced to both this and the cerebro-spinal systems, as mentioned by Brachet and others; or again, that, as we are told by Cowan and a few more, the gradual development of the former of these systems,—its early existence in the animal scale, and its close association with the necessary organic functions, would tend to the conclusion that it is not susceptible to intermittence, and that all intermittent diseases must be regarded as either primary or secondary affections of the cerebro-spinal system alone, need not form the subject of special examination in this place. It will be sufficient to remark that the phenomenon is an attribute of some portion of the nervous system, and that though periodic fevers are not at first, or indeed at any time, exclusively located in this system, yet the fact of these fevers being marked by periodic intermissions or remissions, shows that the latter is implicated in a particular manner in the chain of morbid actions constituting the disease.

All conditions of the economy, all morbific or internal agencies which are capable of affecting the nervous system, necessarily exercise some influence in furthering the manifestation of phenomena appertaining to that system. But again, we repeat, they cannot by themselves produce the effect, and many of them must rather be viewed as manifestations of the law of the economy on which the phenomenon of periodicity depends, than as influencing agents of its development. More, perhaps, is due to the power of habit, to which, as we have seen, a great part of the agency in the production of the phenomenon is ascribable. Every one is aware of the tendency observable in the animal system to reproduce certain actions, simply because they have been produced before; and to this tendency has been and may be ascribed the repetition of febrile paroxysms. An interesting experiment, performed by M. Brachet, of Lyons, on himself, shows very satisfactorily the influence of this acquired habit, and connects itself with the peculiar phenomena of periodic fever. Towards the end of October, 1822, M. Brachet took a cold bath, at midnight, for seven nights in succession, in the river Saone. On the first occasion, he remained a quarter of an hour in the water; then a full hour at a time. After each bath, he betook himself to a warm bed, and in a short time became affected with considerable heat, followed by copious perspiration, in the midst of which he fell asleep. At the end of the seven days, M. Brachet ceased to repeat this experiment; but what was his surprise at finding, on the following nights, between twelve and one o'clock, that all the phenomena of a true ague fit appeared in due order of succession. As, however, this artificial paroxysm was not very severe, and as he felt quite well during the day, with a good appetite and a healthful performance of all the functions, M. Brachet determined not to interfere with, but to observe, the result. Six times it recurred with great regularity. On the seventh night after he had omitted the baths, he was summoned towards midnight to a woman in labor, at the Red Cross. The rapidity with which he ascended to her house overheated him, and on his arrival, he kept up the heat by exposing himself to the action of a large fire. From that time the febrile phenomena ceased to recur.*

But habit alone, especially in the sense in which it is usually understood, cannot account for the effect. When a person, after exposing himself to the air of an infected locality, experiences a paroxysm of periodic fever, we can scarcely attribute the recurrence of a similar paroxysm the next day, or the day after, to the mere power of habit. Could the organs or tissues affected on the occasion of the first paroxysm, have been so rapidly habituated to the morbid impress as to take on the disease after an interval of a few hours, and to continue doing so at regular times long after? If so, how comes it that in times of general prevalence of the disease so many individuals exhibit this power of habit; while ephemeral attacks of fever, under other circumstances, fail to show evidences of that power, by not being followed by a repetition of the febrile excitement? On the other hand, a repetition of the attack—as, for example, in cases of relapses—though taking place at regular intervals, occur sometimes too long after the first, sometimes weeks or even months, to justify our referring the effect to the exercise of the power of habit. If this power is the controlling agent in ordinary cases of periodic fever, what has become of it during those long intervals? It is almost universally admitted that intermittent fevers of paludal origin are identical in nature with remittents. Now if to the power of habit we are to look for the regular and periodical recurrence of the paroxysm in the former, we must attribute to the same agency the recurrence of the exacerbation in the latter. Is this the probable explanation, in any instance, and especially in those in which the attack has come on soon after a single and momentary exposure to the paludal poison? What time have the affected organs had to become so habituated to the morbid impression, as to take on the same action after a repose of some hours? How comes it to pass, admitting habit to be the controlling

* Observations et Recherches sur les F. Interm.—Archives Générales de Méd. ix, 354–5.

agent, that in some places, and in some seasons, this power exhibits itself most frequently, if not universally, every day, then every other, or every fourth day? How is it, also, that at other places and seasons the effect is produced almost invariably every third, or every fourth, or even every seventh day? Let it not be said that in most cases this habit had already been established at the time of the opening paroxysm; inasmuch as the action of the efficient cause has already been produced several times, though without eliciting a manifestation of morbid effects, and each time has been followed by a movement of reaction. However true this may be, in some instances—and even of this we have no proof—the statement cannot apply to very many others; for in these the attack comes on too soon after exposure and too frequently without any other antecedent exposure, to justify the admission of this latent and unobserved periodic movement of reaction.

In closing these remarks, we cannot but look with favor on the idea that morbid organic periodicity, which, as already said, is an attribute of the nervous system, and purely a nervous phenomenon, manifests itself in virtue of a sudden influx of nervous power on some irritated part, where it becomes concentrated and occasions a momentary sanguine congestion, which in its turn occasions the various phenomena of inflammation, local and general. This nervous excitement next becomes exhausted. Soon after, it again becomes restored to the extent of occasioning another state of excitement similar to the former, and continues to manifest itself with these alternations of repose, so long as the organ which constitutes the starting-point of the paroxysm continues in a morbid state; in other words, while it remains in a state of irritation, or retains the secondary impression of the paroxysm, or rather of the congestion. The parts acquire the habit of repeating the same act, because they remain in a state of stimulation which constitutes a source of attraction to the nervous influence; inasmuch as whatever be the extent of the agency of habit, it cannot be exercised without the intervention of some awakening cause. If the organs remained free from even the slightest deviation from their normal condition, and the true efficient cause of the disease were removed, that agency would not exhibit its influence. It requires for its manifestation the preservation of some degree of morbid derangement in the organ or tissue constituting the seat or starting-point of the phenomena observed. This morbid derangement may not be appreciable to the senses of even the most experienced and keenest investigator, or of the patient himself; but we are bound to conclude that it exists nevertheless. In intermittent fever, the organs affected cannot, during the short space of an ordinary intermission, return to their normal state, after suffering the shock to which they were subjected during the force of the paroxysm. If this restoration ever takes place, the occurrence must be very rare. Intermittence, as Nepple remarks, constitutes the period of repose, or, as it were, of sleep of the nervous system, during which the central organs of organic life having lost, by the fact of a violent reaction, a large share of their irritability, or, as some would say, of their nervous fluid, fall momentarily into a state of debility such as to be deprived of the power of awakening sympathetic actions in various parts of the body. It is during this period that the nervous system recuperates its suspended energies, and reacquires its normal influence, and the paroxysm is produced through the influence of the same agency which had given origin to the first; because the state of the organs in which that influence is located has not been changed. Hence, all the paroxysms may be said to be linked together, or rather, they are all successive symptoms of the same disease. 'Tis true that the latter appears to terminate at the close of each paroxysm; but it will reappear so long as the local derangement of the impaired organs is sufficiently severe to excite morbidly the nervous system, which is itself already in a state of disease.

